# The relationship among EFL learners’ motivational self-system, willingness to communicate, and self-assessed proficiency at an Ethiopian preparatory high school

**DOI:** 10.1016/j.heliyon.2024.e24171

**Published:** 2024-01-07

**Authors:** Merih Welay Welesilassie, Marianne Nikolov

**Affiliations:** a Doctoral School of Education, University of Szeged, Hungary; b Aksum University, Ethiopia; c Institute of English Studies, Faculty of Humanities and Social Sciences, University of Pécs, Hungary

**Keywords:** L2 motivational self-system, L2 willingness to communicate, Self-assessed English proficiency

## Abstract

This study investigated relationships between English as a foreign language (EFL) students' L2 motivational self-system (L2MSS), L2 willingness to communicate (L2WTC) inside and outside the classroom, and their self-assessed English proficiency at an Ethiopian preparatory high school. Data was collected using validated instruments from 609 12th-grade preparatory school students in Ethiopia. Using structural equation modelling (SEM), a hypothesised model was generated and tested. The components of the L2MSS were considered as independent variables, while L2WTC within and outside of the classroom and self-assessed English proficiency were treated as dependent variables. Self-assessed English proficiency was also used as an independent variable to test if it predicted scores on the two L2WTC subscales. The results revealed that, although the mean on the ought to L2 self scale was above average, the means of ideal L2 self and L2 learning experiences were below average. Students reported low levels of L2WTC and self-perceived proficiency in English. The components of the L2MSS in the model demonstrated a statistically significant positive association with each other, as well as with L2WTC in and outside the classroom and self-assessed English proficiency. To be more specific, the L2MSS parts had a statistically significant positive effect on the dependent variables. The only one that was not significant was the path from ideal L2 self to L2WTC outside of school. Self-assessed English proficiency showed statistically significant positive predictive effects on L2WTC within and outside the classroom. The results and implications are critically discussed to inform English educators, students, parents, curriculum designers, and researchers about these interrelationships.

## 1 Introduction

According to Dörnyei and Ryan [1], second language learning (L2) motivation is the “primary impetus to initiate learning, and later, the driving force to maintain the long, often tedious learning process; indeed, all the other factors involved in second language acquisition (SLA) presuppose motivation to some extent” (p. 72). The importance of motivation and how it relates to other aspects of the SLA process has been of interest for decades. Extensive research on motivation for language learning has led to the development of new theories and models, including the construct of willingness to communicate in a second language (L2WTC). L2WTC referes to the willingness to participate in a conversation in L2 with a particular person or group of people at a certain time [2]. To reveal what factors contribute to L2WTC, Macintyre et al.[2] developed a model. Several linguistic, communicative, psychological, and social aspects are hypothesized to affect L2WTC in the model. A prominent trait attributed to L2WTC is motivation [3].

Many empirical studies have investigated the association between L2MSS and a variety of learning characteristics, including anxiety [4,5], and L2WTC [[6], [7], [8]]. Others have examined relationships between L2MSS and L2 achievement [9,10]. Most studies examined how L2MSS and L2WTC were related to objective achievement measures (i.e., school grades and proficiency test scores). However, as Csizér and Dörnyei[11] pointed out, the relationship between motivation and test results or performance is only indirect, since motivation is an antecedent of action rather than achievement. Studies that examine the association between motivation and L2 achievement measures (e.g., course grades) show a misleading linear connection between motivation and learning outcomes because they overlook the intermediary link called motivational drive [11]. Despite calls for more studies, there is a lack of evidence in the literature connecting motivation and L2WTC to subjective self-rated proficiency, indicating a gap in the literature.

In the context of the present study, in Ethiopia, English is a compulsory subject taught in Ethiopian primary schools from grade one to grade eight. Furthermore, English is used as the medium of instruction (MOI) in secondary schools (grades nine through twelve) and postsecondary education (universities). Therefore, it is safe to say that the proficiency of students in English has a substantial influence on their academic results in the Ethiopian educational environment. However, many English students are unsuccessful and reluctant to use the L2 inside or outside the classroom. Although the key roles of motivation and talking to learn are now acknowledged in contemporary L2 instruction, drawing from my personal experience as an English teacher spanning over 12 years, coupled with existing empirical study by Welesilassie and Nikolov [5], it becomes evident that many learners are unmotivated and reluctant to speak in English, despite their awareness of the necessity to do so. Furthermore, since L2MSS and L2WTC are both dynamic and context-specific, their applicability in the multicultural setting of Ethiopia required empirical support. As teachers and decision makers try to improve English programs, it is important to find the reasons why English teaching in public education is not as successful as expected. Therefore, it is a necessry to collect data on the role of motivation in the L2 learning process as well as to examine why Ethiopian students are reluctant to speak in English. In line with these educational needs, the aim of this study is to examine the relationships between L2MSS, L2WTC within and outside the classroom, and self-assessed English proficiency in the Ethiopian educational context, where no previous research has been conducted on these constructs. The findings are expected to shed light on how L2 instructors can offer context-specific pedagogical assistance based on the experiences of their students and help increase their motivation and willingness to communicate and practice within and outside the classroom.

## 2 **Review of the literature**

In this overview, we offer a critical analysis of some of the most prominent L2 motivation theories and empirical investigations in EFL contexts. Our aim wa to discuss the constructs of L2 motivation and L2WTC in various contexts and their relationships.

### 2.1 L2 Motivational Self-System (L2MSS)

Although motivation has been in focus in the fields of psychology and education for decades, it has only recently become a key area of study in SLA. As the driving force underlying the process of learning a new language, motivation is an essential factor in L2 learning [1,3,12]. Motivation is a crucial factor in determining how individuals approach their goals, persist in their efforts, and make decisions. Various theoretical frameworks have been proposed over the past five decades to better understand the nature of L2 motivation. One of the most noteworthy theories regarding L2 motivation is the L2MSS theory, which was developed by Ref [3]. This theory incorporates concepts such as motivational transformation, self-regulation, imagined (ideal) selves, and the cultivation of individual motivation within sociocultural contexts.

L2MSS has two main parts: self and context. The self part is split into two sections: the ideal L2 self embodies all the qualities one desires to possess, and the ought-to L2 self encompasses the traits that one should have to meet obligations and expectations as well as to avoid unfavourable consequences [3,12]. These two selves were formulated based on two leading theories: possible selves and self-discrepancy theory.

The theory of Possible Selves , first proposed by Markus and Nurius [13], posits that everyone has a vision of their potential future selves, encompassing both desirable and undesirable versions. The desired selves represent the ideal future identity, while the undesirable selves represent aversive future identities [13]. This theory suggests that people are driven to pursue their desired selves and avoid their feared selves, influencing their choices, actions, and aspirations. Furthermore, the theory proposes that a person's self-concept comprises not only their present self, but also their future self.

According to the Self-Discrepancy theory, developed by Higgins [14], individuals possess multiple self-representations, consisting of the actual self, which refers to how they perceive themselves, the ideal self, which reflects the person they ideally wish to become, and the ought self, representing the person they believe they ought to be based on external expectations [14]. The existence of a divergence between these various self-representations, such as a discrepancy between the actual self and the ideal self or ought self, may produce emotional discomfort and motivate individuals to lessen the gap. This theory proposes that individuals are inspired to align their actual self with their ideal and ought selves, and the degree of incongruity may impact their emotional well-being and motivation. The two theories, possible selves and self-discrepancy theory, offer valuable insights into comprehending motivation and self-perception. They have been widely used in various fields such as psychology, education, and organizational behavior. By understanding the concept of L2MSS and how these theories shape the two selves, individuals can gain a better understanding of their motivations.

In the realm of L2 learning, the concept of "context" refers to the learning experiences that are related to the current situations and circumstances in which the learners find themselves. This component is crucial to understanding how the learning experience can be influenced by various factors such as the teaching methods employed in the classroom, the curriculum, and the presence of significant others such as peers and parents [15]. The context provides a framework through which the learners can understand the relevance and importance of the L2 to their lives and the world around them. It can also help motivate students and enhance their desire to learn the L2, which in turn can lead to better learning outcomes. Thus, it is important to consider the role of context in L2 learning and to create an environment that is conducive to effective learning.

### 2.2 L2 willingness to communicate (L2WTC)

Talking to learn [16] is a theory that has garnered a lot of support in SLA research. Current language teaching pedagogies are shifting toward a conversational approach, encouraging students to participate in meaningful conversations to practice their L2 and gain confidence. The construct of WTC in L2 was first characterized as a consistent personality trait in L1. When seen through the lens of L2, WTC is distinctive because, in L2 contexts, the level of learning of L2 skills and their motivation can vary substantially; these can impact the how well they can and want to communicate effectively. Thus, L2WTC weighs the merits and effects of the state (situational-based factor) and trait (personality-based factor) [2]. Accordingly Macintyre et al. [2], adapted WTC to L2 settings and conceived L2WTC as ready-to-join a L2 conversation at a given moment with certain individuals. They provided a model comprising what leads to L2WTC so that it can be comprehended in depth. Therefore, they proposed that L2WTC is the result of a combination of proximal and distant factors, including linguistic, psychological, communicative, social, and environmental aspects [2].

### 2.3 Studies on relationships between L2MSS, L2WTC, and L2 proficiency

Understanding the complex interplay between L2MSS, L2WTC, and self-perceived English proficiency is an area of great significance in the field of language learning research. Over the years, there have been several studies that have delved deeper into the relationships between these variables in diverse contexts around the world. From Japan to Ethiopia, Hungary to Indonesia, Iran to China, and Korea to many other countries, researchers have explored the intricate dynamics that govern these factors, shedding light on their impact on language learning and acquisition.

Numerous studies have been conducted on the correlation between components of L2MSS , including the ideal L2 self, ought-to L2 self, L2 learning experience, as well as L2WTC within and outside the classroom. For example, a study by Sadoughi & Hejazi [17] explored how the L2MSS components and L2 anxiety contribute to the prediction of L2WTC among Iranian EFL learners in the classroom. The results of the correlation study showed that the WTC in the classroom was significantly and positively related to the ideal L2 self, the ought-to L2 self, and the L2 learning experience. The ideal L2 self was positively correlated with ought-to L2 self and L2 learning experience. Similarly, L2 learning experience showed a positive relationship with ought-to L2 self. Both the L2 learning experience and the ideal self directly and positively influenced WTC in the classroom. The ideal self and learning experience had a negative impact on L2 anxiety, while ought-to self exerted a positive effect. L2 anxiety, in turn, was found to have a negative impact on WTC. Furthermore, in a study conducted by Peng [4] in China; the author reported high levels of L2WTC of students inside and outside of the classroom and the three elements of L2MSS. The study found statistically significant positive relationships between the three variables of L2MSS. Additionally, the study found that L2WTC in the classroom had a positive but weak association with the ideal L2 self and L2 learning experience, while L2WTC outside of the classroom had a positive but barely significant link with the ideal L2 self and L2 learning experience. However, the ought to L2 self had no statistically significant relationship with either L2WTC in or outside the classroom. The relationship between L2WTC in both contexts was positive but weak. The study also found that the ideal L2 self was directly influenced by the L2 learning experience and ought to L2 self, and that the L2 learning experience directly predicted L2WTC inside the classroom. These research findings provide compelling evidence of the complex links among L2 anxiety, the L2MSS elements (such as the ideal L2 self, ought-to L2 self, and L2 learning experience), and L2WTC, both inside and outside the classroom. The ideal L2 self and L2 learning experience consistently displayed favorable associations with WTC, underscoring its significance in fostering communicative actions. In addition, the adverse impact of L2 anxiety on WTC underscores the need to adress and alleviate language-related anxieties to enable successful communication.

In a study conducted in the Korean EFL context, Lee and Lee [7] investigated how the L2MSS impacts L2WTC. Their findings indicated a moderate ideal L2 self, a low ought to L2 self, and low levels of L2WTC both in and out of the classroom. They also discovered significant positive relationships between the ideal L2 and the ought to L2 self, as well as L2WTC in both settings. Ought to L2 self was found to be positively and significantly related to L2WTC in both settings, with the strongest relationship being between L2WTC in and out of the classroom. The study also revealed that L2WTC in both settings was significantly and positively predicted by the ideal L2 self and the ought to L2 self. According to a study conducted by Zhou [8] in Southwest China, the participants' ideal L2 self had the highest mean value in L2MSS, with the ought-to self having the lowest. The mean value of L2WTC fell in the upper-middle range. Furthermore, the results revealed significant positive correlations between the three factors in L2MSS, with all three elements positively related to L2WTC both in and out of the classroom. However, only the ideal self and L2 learning experience significantly impacted L2WTC. The findings of these two studies provide comprehensive evidence of the relationships between L2MSS and L2WTC. The ideal L2 self and L2 learning experience consistently demonstrated positive associations with WTC both inside and outside the classroom, highlighting their important predictive effects. However, the ought-to L2 self had limited predictive effects on WTC.

Another, two studies, one by Yashima et al. [18] focussing on Japanese high school students and the other by Nagy [6] on advanced Hungarian English learners, found a strong correlation between perceived communication skills and L2WTC, both inside and outside the classroom. The Japanese study revealed that self-perceived communication competence was the best predictor of L2WTC inside and outside the classroom. According to the findings of the Hungarian study, there exists a notable relationship between the perceived communication competence of the tudents and their L2WTC outside the classroom. The study further highlights that students' own perception of their communication skills plays a vital role in boosting their willingness to communicate outside the classroom settings. Both studies highlighted the significant role of self-perceived competence in influencing the LTWTC inside and outside the classroom.

Another study conducted by Subekti [9] examined the connection between L2MSS and academic achievement in undergraduate students from Indonesia. The participants displayed a high level of motivation for L2 learning, with their ideal L2 self-rating the highest among the three components of L2MSS. The correlation between the three components of L2MSS and EFL achievement was found to be insignificant. The author concluded that despite the experts’ claims that L2MSS predicts L2 achievement, participants' L2MSS did not significantly predict EFL achievement. On the other hand, Roshandel et al. [19], explored the relationship between motivation and self-efficacy among Iranian EFL students. After conducting correlation and regression analyses, the results showed that there were modest to moderate positive associations between self-efficacy and the two selves: ideal L2 self and ought-to L2 self. In a similar vein, Shih and Chang [10], also reported that the ideal L2 self and L2 learning experience significantly impacted self-efficacy among Taiwanese high school students. The results showed positive associations between self-efficacy and various factors, including ideal L2 self, ought-to L2 self, and learning experience. These findings indicate that motivation is a precursor to action, rather than achievement, showing an indirect relationship between motivation and achievement.

Finally, in the Ethiopian EFL context, Welesilassie and Nikolov [5] explored the perceived L2MSS and its relationship to L2 anxiety among university students. The study found that students rated their L2 learning experience and the ideal L2 self as low, while their perception of the ought to L2 self was high. The strongest significant relationship was found between the ideal L2 self and the L2 learning experience. There was also a weak but statistically significant link between debilitating anxiety and the two component of L2MSS , which were the ideal L2 self and L2 learning experience.

In conclusion, the findings of these studies have revealed various associations between students’ language learning achievement, L2MSS, and L2WTC. The observed disparities may be ascribed to the utilization of diverse evaluation instruments that emphasized objective indicators of L2 competence. It is imperative to acknowledge that the association between motivation and objective test scores is of an indirect nature, given that motivation serves as a precursor to action rather than exerting a direct influence on achievement. Studies that exclusively focus on investigating the correlation between motivation and objective indicators of achievement, such as academic performance, may inadvertently disregard the crucial mediating element known as motivational drive [11]. Consequently, this oversight may result in a potentially misleading implication of the relationship as a linear connection. To effectively adress these disparities, it is imperative to integrate self-rated subjective proficiency measures that encompass a wide range of student skills.

Although studies have been conducted on the influence of L2MSS on L2WTC and self-assessed English proficiency, most of the research has been limited to the classroom context. However, it is necessary to conduct a more thorough and evidence-based analysis to investigate the L2WTC and self-assessed English proficiency in real-world contexts beyond the boundaries of the educational setting. This is crucial due to the higher probability of experiencing dynamic emotional states associated with L2 communication in everyday interactions, as opposed to a structured educational setting.

Moreover, it should be noted that most of the L2WTC research has focused on the assessment of oral communication skills. However, it is important to note that within the context of Ethiopian foreign language education, a distinct emphasis has been documented on the cultivation of reading comprehension, writing proficiency, and grammatical competence. In contrast, less attention is given to listening comprehension and speaking abilities. Hence, it is imperative to study L2MSS and its impact on L2WTC and self-assessed English proficiency, which encompass all four language skills, within specific cultural and educational settings like Ethiopia. Therefore, this study aims to bridge the identified gaps outlined in the current literature.

Finally, conducting research on the interplay between L2MSS, L2WTC, and self-perceived English proficiency in the Ethiopian context is of utmost importance. Surprisingly, this field of study has not yet been explored well. The dynamic nature of these variables, which are influenced by time, topic, and context, underscores the value of conducting research in multilingual and multicultural settings such as Ethiopia. Such research could provide a more in-depth understanding of L2 learning and contribute significantly to the existing literature on this topic.

### 2.4 The study

The present study aimed to investigate the relationships between the components of L2MSS, L2WTC inside and outside the classroom, and self-assessed English proficiency in a group of 12th grade EFL Ethiopian students. Additionally, it tested the causal relationships among the variables under investigation. The following research questions were posed.

1. How was the Ethiopian students' L2MSS, L2WTC inside and outside the classroom, and self-assessed English proficiency characterized?
2. How did the Ethiopian students' L2MSS, L2WTC inside and outside the classroom, and their self-assessed proficiency in English relate to one another?
3. What was the predictive effect of students' L2MSS on their self-assessed English proficiency and L2WTC inside and outside the classroom?

### 2.5 The proposed model

The current investigation aimed to understand the relationships between L2MSS, L2WTC, and self-perceived English proficiency of high-school students in the Ethiopian EFL context. The initial model was constructed by integrating six variables.: ideal L2 self, ought to L2 self, L2 learning experiences, L2WTC inside the classroom, L2WTC outside the classroom, and self-assessed English proficiency. The model specifications were based on the theories and empirical studies presented in the literature review.

Based on the evidence offered in Refs. [7,17], direct and significant positive paths were drawn from the ideal L2 self and ought to L2 self to L2WTC inside and outside the classroom. We predicted additional direct and significant positive paths from L2 learning experiences to L2WTC in and outside of the classroom, also confirmed by Refs. [4,8,17]. In the realm of EFL education in Ethiopia, students are motivated to acquire English language skills for various reasons, including future career prospects, academic success, and personal growth. Therefore, it is reasonable to assume that by cultivating the L2 selves, working towards it, and fostering positive learning experiences, students will achieve proficiency in both formal and informal settings. As learners envision themselves as confident and competent English speakers in their future professions, perceive English as a means to unlock better job opportunities, and enjoy a positive learning journey, they will be more inclined to communicate fluently and confidently with others in English.

We anticipated that self-assessed proficiency positively and directly influenced L2WTC inside and outside the classroom, substantiated by the findings of previous inquiries, such as [6,18]. Accordingly, two hypothetical paths were proposed that lead from self-assessed English proficiency to L2WTC within and outside of the classroom were proposed. The rationale underlying these proposals was that students would be more ready to communicate in L2 if they believed more strongly that they could communicate in English. In Ethiopia, it is common knowledge that many students experience shyness and anxiety when speaking English. However, students who have confidence in their language abilities are more likely to use English in various settings. This underscores the significant influence of self-perception on a student's readiness to communicate effectively in English. By fostering a positive mindset and providing the appropriate support, Ethiopian students can overcome their anxieties and become willing to communicate in the English language.

**Fig. 1:**
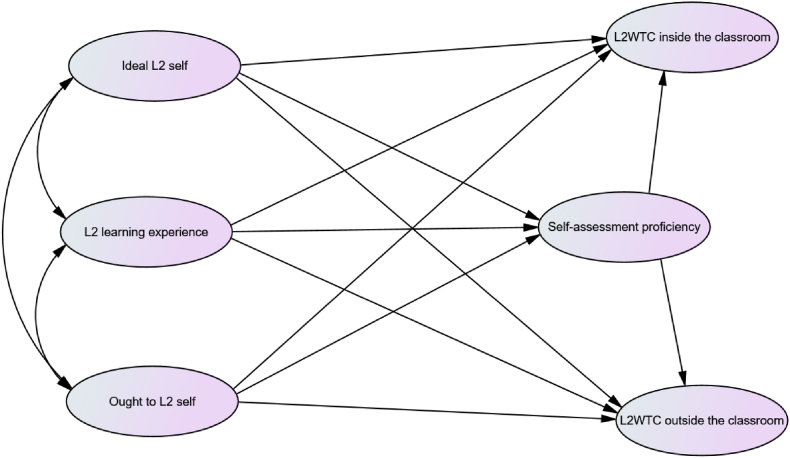
The hypothesized model.

Finally, we hypothesized a direct and significant positive path from the ideal L2 self, the ought L2 self and L2 learning experiences to self-assessed proficiency in English. These paths were based on evidence from earlier empirical studies [10,19]. In the context of EFL instruction in Ethiopia, students who aspire to become proficient English users, believe they ought to improve their language skills, and have positive learning experiences, tend to exhibit heightened motivation toward participating in language learning activities. This, in turn, leads to increased exposure to the English language , more opportunities to practice, and an elevated sense of proficiency, ultimately influencing their self-assessed English language competence. Fig. 1 demonstrates the proposed model.

## 3 Method

### 3.1 Research design

To address the research questions and test the hypotheses, we employed a quantitative cross-sectional research design [20]. We designed and validated the instruments in September 2022. After that, questionnaire data were collected in early November 2022. The descriptive and correlational coefficients were then determined using the statistical software SPSS - version 25.0. The suggested model was tested using SEM using IBM SPSS AMOS version 23 statistics. Finally, the statistical findings were analyzed, and their educational implications were proposed.

### 3.2 Setting and participants

The study was carried out at the Mizan-Aman Preparatory School, located in the southwestern regional state of Ethiopia, during the first semester of the academic year 2022–2023. Participants were enrolled in their 12th year of secondary education. The students pursued their academic studies in English, using it as both the language of instruction and the subject of their studies. The research focused on this group of learners owing due to their notable educational milestone of transitioning from secondary school to tertiary education. Within the educational landscape of Ethiopia, it is imperative to acknowledge the profound importnace attributed to the 12th grade. This stage of academic progression represents a critical juncture, symbolizing a moment of utmost importance and relevance. Therefore, the findings of the present study are expected to provide important insights into students' L2MSS, L2WTC, and perceived communication abilities and may help identify the implications of their motivational profile on their L2WTC. Studying 12th grade students can help stakeholders at higher education institutions understand the need of their novice students and plan their teaching based on the results of this study. The school had a total of 652 students, with 314 females and 338 males. The study utilized a purposive sampling technique [20]. We engaged all students in the school to gain comprehensive insights into the phenomena under investigation. Using this method made it easier to come up with generalizations about the target population, which made it less likely that any possible data points would be missed.. Of the total population, 609 students (352 men and 257 women) freely agreed to participate in the investigation and completed the questionnaire within the allotted time. The age range of the participants was 18–23 (M = 20.6, SD = 0.72).

### 3.3 Instruments

The survey questionnaire was designed and administered following the guidelines suggested byDörnyei and Taguchi [21]. There are four sections in the questionnaire. The first section focusses on the students' background (i.e., age, gender,grade). The students provided information without disclosing their names or identification numbers. Section two includes the three components of the L2MSS, which we adapted from Welesilassie and Nikolov [[5] as it had been validated in the Ethiopian context and worked well. These were: (1) ideal L2 self, 10 items measuring learners' ambitions and aspirations about using English in the future; (2) ought to L2 self, 10 items assessing the degree to which students felt they were required to study English and speak it well; and (3) L2 learning experience, 12 items measuring how students felt about aspects of their immediate educational setting, including instruction and classroom dynamic. Section three comprised the adapted L2WTC inside [22] and outside [6] the classroom. Originally, the L2WTC inside the classroom scale had 27 items, while the L2WTC outside the classroom comprised 16 items. Section four consisted of items related to the self-assessed English proficiency of the students. We used the Common European Framework of References for Language (CEFR) as a baseline to categorize students' English skills. The CEFR descriptors categorize learners' abilities at six distinct levels of language proficiency: A1-A2 (basic user), B1–B2 (independent user), and C1–C2 (proficient user) [23]. In Ethiopia's educational system, there is currently no standardised Common European Framework of Reference (CEFR) in use to measure students' proficiency in English. Therefore, it is difficult to determine their exact level of competence. The only way to assess their English language proficiency is through final tests and national examinations. To address this gap, we have developed our own self-assessed English proficiency evaluation based on three criteria. First, we have collected specific objectives for each chapter from the preparatory textbooks (grades 11 and 12) to understand the core competencies that students are acquiring. Second, we have analyzed the final and national exams to determine whichcompetencies are being measured. Finally, based on my 13 years of experience teaching English in various higher educational institutions in Ethiopia, I have related this information to CEFR-2020. After this process, we have categorised students as "basic users" (A1 and A2) of English. The eleven items were then constructed in the form of 'can do' statements for students to decide how well they thought they could do things on a six-point Likert scale. The scale included items related to reception (listening and reading skills), interaction (spoken and written interaction), and production (spoken and written production). Eleven items on self-assessed English proficiency were included.

Before conducting a pilot study, we reduced the number of items in the questionnaire. To achieve our goals and link them with the FL learning experiences of Ethiopian high school students, we eliminated and adjusted some items. As a result, the number of items in the L2MSS related to the L2 learning experience decreased to ten. Furthermore, the L2WTC items inside and outside of the classroom were reduced to eleven and eight, respectively. We also removed two items related to self-assessed English proficiency. All items were then translated into Amharic, the participants’ first language, with the help of an expert from Addis Ababa University in Ethiopia and two additional experts from Mizan-Tepi University in Ethiopia. The translators were senior Amharic and English language and literature professors.

After completing the translation, we piloted the instruments in September 2022 with four teachers and twelve students. In the pilot research, we examined how well the items worked, and met criteria of appropriateness, ease of use, and effectiveness. Based on information gained in the pilot, we eliminated six items from the L2WTC within the classroom scale and three from the L2WTC outside the classroom scale. Some items needed to be combined (for example, the items "I am willing to talk in English with an English-speaking friend while standing in line." and "I am willing to talk in English with an English-speaking girl/boyfriend." were merged into "I am willing to talk in English with an English-speaking friend while standing in line". In a similar vein, we eliminated two items from the self-perceived scale because one was found to be too easy (i.e., "I can tell people their name and ask other people their names") and the other was too challenging ("I can make personal online comments about experiences, emotions, and events"). Furthermore, three items were eliminated from each component of the L2MSS due to their level of complexity. Accordingly, seven ideal L2 self-items, seven ought to L2 self-items, seven L2 learning experience items, five L2WTC inside-the-classroom items, five L2WTC outside-the-classroom items, and seven self-assessed English proficiency items were included in the final questionnaire.

### 3.4 The procedure of data collection and analysis

Data collection for the main study began by requesting permission to conduct the research from the school's administration. Once permission was granted, we contacted the teacher in charge of organising the classes; then, we collected data in early November 2022. The participants received explanations of the objectives, benefits, and problems of maintaining data security from the assigned teacher (in-person) and the first author (online). The questionnaire was answered by students who had signed their consent forms. On average, respondents spent 30 min answering the items. Data from participants was collected and coded and then analysed using IBM SPSS 25 and IBM SPSS AMOS 23 statistical software. The measurement model and the structural model comprised variables of the SEM model. The method of maximum likelihood was utilised to arrive at an accurate estimation of the parameters. The literature review revealed certain theoretical issues and served as the basis for the creation of the initial measurement model. Subsequently, the latent variables were incorporated into a comprehensive structural model. Using the metrics suggested in the SEM literature, the overall fit of the model was assessed. Descriptive statistics such as minimum, maximum, mean, and standard deviation were developed to better understand the students' L2MSS, and L2WTC inside and outside the classroom, and their self-assessed English proficiency. Pearson product-moment correlation coefficient (r) was calculated to determine the existence and significance of the correlations among the variables.

### 3.5 Ethical considerations

The study was carried out in accordance with the requirements of University of Szeged Ethics Committee (IRB) r and official permission to proceed was obtained. We also obtained permission to collect data from Mizan-Aman preparatory school in Ethiopia's southwest region. Each participant was given an oral and written description of the objectives, as well as information that their participation was fully voluntary and that they could withdraw their agreement at any time. All data was handled discreetly and anonymously.

## 4 Results

In this section, we present the findings. Two steps of data analysis were performed: confirmatory factor analysis (CFA) was used to evaluate the measurement models for the six variables, and structural equation modeling (SEM) was used to test the structural model. Furthermore, the descriptive and correlational results as well as the predictive effects of the variables under study are presented, respectively.

### 4.1 Testing the measurement model

Before merging the six measurement models into a complete structural model, we first evaluated each measurement model independently to test the fit of the model. By looking at the internal consistency reliability (Cronbach's α), the reliability of the instrument was also tested. All measures were greater than 0.6, which is considered reliable [21]. SEM obtained using AMOS was utilised to test the measurement model. Using the Chi-square (X^2^) value – X^2^ (X^2^/df), and other goodness-of-fit criteria, the suitability of the measurement models and the subsequent entire structural model was assessed. A model is deemed acceptable if the value of X^2^ (X^2^/df) is less than five. Numerous other fit indices were considered: the Root Mean-Square Error Approximation (RMSEA), Goodness-of-Fit Index (GFI), Tucker and Lewis index (TLI), and the Comparative Fit Index (CFI). For a model to have adequate goodness of fit, the GFI, TLI, and CFI should be greater than 0.90 [24]. The RMSEA should be in the range of 0.05–0.08. Table 1 shows that the reliability and fit indices for the model were within an acceptable range. Some items were taken out of the measurement models because the factor loading was either larger than 1.00 or less than 0.30. Items 4 and 9 in the ideal L2 self, 11 and 18 in the ought to L2 self, 21, 22, and 23 in the L2 earning experience items, and 38, 39, and 41 in the self-assessment English competency scales were thus eliminated.

**Table 1:** Fit indexes for the measurement models of the six variables.

Variable Scales	X^2^	df	X^2^/df	CFI	TLI	GFI	RMSEA	α.
Ideal L2 self	21.05	5	4.21	.98	.97	.98	.07	.81
L2 Learning experience	24.719	9	2.74	.98	.97	.98	.05	.81
Ought to L2 self	22.612	5	4.52	.99	.98	.98	,07	.93
L2WTC inside the classroom	21.893	5	4.37	.98	.97	.97	.04	.92
L2WTC outside the classroom	13.310	5	2.66	.99	.99	.99	.03	.83
Self-assessed proficiency	18.780	5	3.75	.98	.97	.98	.06	.82

**Testing the full structural model**

According to the preliminary analysis, the proposed model offered an accepted explanation for the observations. In contrast to what was anticipated, it revealed that the route that led from the ideal L2 self to L2WTC outside the classroom was not significant in the final model. Thus, it was deleted so that the model could be more condensed. Finally, the model matched the data quite well (i.e., x^2^ = 894.133; df = 391; x^2^/df = 2.28; GFI = 0.91, TLI = 0.94, CFI = 0.95, and RMSEA = 0.04).

**RQ-1:** How could students’ L2MSS, L2WTC inside and outside the classroom, and self-assessed English proficiency be characterized?

Presented in Table 2 is a descriptive analysis of the population, including the minimum, maximum, mean, and standard deviation on the three components of L2MSS (i.e., ideal L2 self, ought to L2 self, and learning experience) L2WTC inside and outside the classroom, and the self-assessed English proficiency.

**Table 2:** Descriptive analysis of the ideal L2 self, the ought to L2 self, L2 learning experience, L2WTC in and outside class, and self-assessed proficiency in English.

Variables	Minimum	Maximum	Mean	Std. Deviation
Ideal L2 self	1.00	4.20	2.61	.78
L2 learning experience	1.00	4.00	2.25	.70
Ought to L2 self	1.00	6.00	4.03	1.46
L2WTC inside classroom	1.00	6.00	2.75	1.16
L2WTC outside classroom	1.00	4.50	2.58	.92
Self-assessment proficiency	1.00	4.80	2.35	.89

According to data in Table 2, the overall mean scores of the student in the three components of the L2MSS ranged from 2.25 to 4.03 points. Students’ L2 learning experience, the perception of their current learning situation, and the degree to which they were satisfied with their L2 learning experience were the lowest (M = 2.25, SD = 0.70), followed by their ideal L2 self (M = 2.61, SD = 0.78), which, according to Ref. [5], is one of the most powerful and effective motivational factors.

L2WTC was also low both inside the classroom (M = 2.75, SD = 1.16) and outside the classroom (M = 2.58, SD = 0.92), showing that students were unwilling to participate in English conversation in their classes or beyond them. Similarly, students’ self-assessed proficiency was low (M = 2.35, SD = 0.89), indicating that the students believed they could not communicate in English either in instructional or in informal situations.

**RQ-2:** How did students’ L2MSS, L2WTC inside and outside the classroom, and their self-assessed English proficiency relate to one another?

Table 3 presents the relationships among the participants' self-perceived L2MSS, L2WTC inside and outside the classroom, and self-assessed English proficiency. To find out if any correlations were significant, the pearson product-moment correlation coefficient (r) was computed. We found weak to moderate statistically significant relationships across the six variables.

**Table 3:** Correlation analysis of the ideal L2 self, the ought-to L2 self, L2 learning experience, L2WTC in and outside class, and self-assessed proficiency in English.

Variables	1	2	3	4	5	
1. Ideal L2 self						
2. L2 learning experience	.59**					
3. Ought to L2 self	.52**	.42**				
4. L2WTC inside classroom	.48**	.43**	.25**			
5. L2WTC outside classroom	.41**	.43**	.50**	.37**		
6. Self-assessment proficiency	.46**	.40**	.40**	.52**	.35**	

Note: *p < .05, **p < .01.

According to Pearson correlation coefficients, the three components of L2MSS were positively and significantly correlated with each other. Both the L2 learning experience (r = 0.59, p < .01) and the ought to L2 self (r = 0.52, p < .01) exhibited a moderate statistically significant association with the ideal L2 self. The L2 learning experience showed a moderate statistically positive association with the ought to L2 self (r = 0.42, p < .01).

The two L2WTC subscales (in and outside the classroom) had moderate statistically significant positive associations with one another (r = 0.37, p < .01). L2WTC inside the classroom showed a weak but significant positive link with ought to L2 self (r = 0.25, p < .01), and a moderate significant positive relationship with ideal L2 self (r = 0.48, p < .01) and L2 learning experience (r = 0.43, p < .01). Similarly, L2WTC outside of the classroom showed a moderate positive link with ought to L2 self (r = 0.50, p < .01), ideal L2 self (r = 0.41, p < .01), and L2 learning experience (r = 0.43, p < .01).

The self-assessment of English proficiency exhibited a statistically significant positive connection with both L2WTC subscales: within the classroom (r = 0.52, p < .01) and outside of the classroom (r = 0.35, p < .01). Additionally, self-assessment of English proficiency had a modest statistically significant positive relationship with ideal L2 self (r = 0.46, p < .01), L2 learning experience (r = 0.40, p < .01), and ought to L2 (r = 0.40, p < .01), respectively.

According to the results detailed so far, the three variables of the L2MSS and the two factors of L2WTC are moderately associated with each other and with self-assessed English proficiency. However, his does not imply that the three L2MSS variables significantly predict or influence the two L2WTC factors or self-assessed English competenccy. SEM analysis is required to evaluate causal links among variables, since correlational analysis illuminated only significant relationships among the variables; however, it did not reveal how they impacted each other.

**RQ-3:** What was the predictive effect of students’ L2MSS on their self-assessed proficiency in English, and L2WTC inside and outside the classroom?

**Fig. 2:**
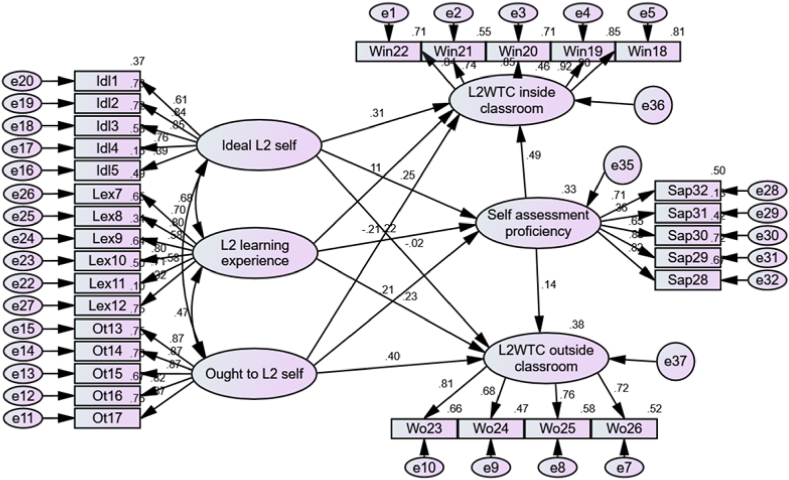
Full structural equation model.

A regression analysis was performed on each of the dependent variables, including self-assessed English proficiency, L2WTC inside, and L2WTC outside the classroom. The self-assessed English proficiency contruct had an R^2^ value of 0.332, L2WTC inside the classroom 0.460, and L2WTC outside the classroom 0.380, respectively. To put it differently, the variances of the endogenous variables that were explained in this model were as follows: 33.2 % for self-assessment proficiency, 46.0 % for L2WTC within the classroom and 38.0 % for L2WTC outside the classroom. Fig. 2 is an illustration of the model.

Next, to determine the impact of each independent variable on the dependent variables, coefficients were also calculated. As shown in Table 4, ideal L2 self (β = 0.24, t = 3.47, p < .001), L2 learning experience (β = 0.22, t = 3.61, p < .001), ought to L2 self L2 (β = 0.20, t = 4.12, p < .001) all exhibited statistically significant positive predictive impacts on self-assessed English proficiency. Positive significant predictors for the L2WTC in the classroom were ideal L2 self, (β = 0.31, t = 4.64, p < .001), L2 learning experience (β = 0.11, t = 1.99, p < .001), ought to L2 self L2 (β = 0.21, t = −4.46, p < .001), and self-assessed proficiency in English (β = 0.49, t = −9.55, p < .001). L2WTC outside of the classroom was positively predicted by L2 learning experience (β = 0.22, t = 3.64, p < .001), ought to L2 self L2 (β = −0.40, t = −7.55, p < .001), and self-assess proficiency (β = −0.13, t = −2.68, p < .001).

**Table 4:** Standardized direct effects for the structural model.

Predicted variable	Predictor variable	β	t-value	P-value
Self-assessment proficiency	Ideal L2self	.247	3.476	***
	L2 learning experience	.224	3.619	***
	Ought to L2self	.208	4.124	***
R^2^		.333		
L2WTC in the classroom	Ideal L2 self	.314	4.640	***
	L2 learning experience	.109	1.990	***
	Ought to L2 self	210	4.636	***
	Self-assessment proficiency	.489	9.557	***
R^2^		.460		
L2WTC outside the classroom	L2 learning experience	.228	3.642	***
	Ought to L2 self	.402	7.550	***
	Self-assessed English proficiency	.138	2.688	***
R^2^		.380		

R^2^ = correlation coefficient.

## 5 Discussion

This research aimed to investigate the relationships among the components of L2MSS, L2WTC within and outside the classroom, and self-assessed English proficiency. Additionally, the predictive effects of the independent variables on the dependent variables were examined using SEM. Here, we provide a critical analysis of the findings by contrasting them with the results of earlier empirical and theoretical studies.

RQ1 How was the Ethiopian students' L2MSS, L2WTC inside and outside the classroom, and self-assessed English proficiency characterized?

The first objective of the study was to describe participants' L2MSS, L2WTC within and outside the classroom, and their self-assessed English competence. For the descriptive analysis, the result for the ideal L2 self was the lowest. This finding was in line with the findings of Welesilassie and Nikolov [5] who reported a low to moderate ideal L2 self, and claimed that students were moderately motivated to envision themselves as capable English speakers. However, it is not aligned with research by Refs. [4,8,9] who reported that students' ideal L2 self was high and claimed that the desire to learn English derived mainly from the vision of the respondents a better self in the future. The ideal L2 self is preoccupied with the internalised goal of becoming a proficient user of English. This idea is based on the premise that motivation increases when people endeavour to bridge the gap between their existing and potential selves. However, the Ethiopian students in our studywere not enthusiastic about the prospect of using English in real-world contexts. They had hardly any interest in working or studying abroad, no intention of communicating with others in English, and no plans to use English in a professional capacity. These results may be attributed to the fact that Ethiopian students, particularly those in the transition years between high school and university, are anxious, dissatisfied with their learning experience, and motivated by fear of failure and external pressure [5]. Students with low ideal L2 selves are more likely to experience increased anxiety arousal than those with a higher ideal L2 self [17] because the latter are more likely to adjust their goals to match the L2 related traits predicted in L2 usage scenarios. In addition, students’ ideal L2 self may decrease due to the anxiety caused by the fear of failure and external pressure represented in ought to L2 selves [4].

Our study found that the level of satisfaction with L2 learning experience in Ethiopia was low, which was consistent with the findings of Welesilassie and Nikolov [5], while contrasting with the high levels of L2 learning satisfaction reported by Peng [4] in China and Subekti [9] in Indonesia. The factor of learning experience is centred on the students’ perceptions of their present learning environment and their level of satisfaction with their L2 learning experiences. Although many researchers such as [1,15] emphasized the importance of L2 learning experiences (i.e., enjoyment) in building long-term motivation, Ethiopian students were unhappy with their English learning experiences. According to Welesilassie and Nikolov [5], various elements such as uncomfortable classroom atmosphere, poor teaching and learning conditions, time limits for performing pair and group activities, and a high student-to-teacher ratio may lead to dissatisfaction with English learning in the Ethiopian context.

Unlike the ideal L2 self and L2 learning experience, the mean for ought to L2 self was high. The term ought to L2 refers to a person's perceived responsibility to avoid negative consequences. Our research has revealed that students from Ethiopia are mostly motivated to learn English due to external factors that include societal pressure, expectations from parents, teachers, and peers, and the prospect of better career opportunities and grades. These external factors play a significant role in shaping students' motivation to learn English, rather than their genuine interest. This highlights the cultural and social norms prevalent in Ethiopia, where the ability to speak, English is linked to higher social status and success, making it a desirable skill. Furthermore, English proficiency is viewed as a crucial factor in achieving success in both academic and professional realms by many parents, teachers, and peers. This cultural and social context reinforces the extrinsic motivation of students to learn English. These findings corroborate the results documented by previous studies [4,5], that underscored the underlying reasons for students to learn English as the fear of negative consequences and external expectations and pressure. On the contrary, the outcomes of the present study were at variance with the findings of [7,8], who reported a low level of ought to L2 self.

According to the study, high school students in Ethiopia displayed a low inclination to communicate in English, regardless of whether they were in or out of the classroom. This suggests that students lacked the confidence to employ all four language skills. These findings were consistent with a previous study such as Subekti [9] that also revealed minimal levels of communication both inside and outside the classroom. However, they contradicted with the findings reported by (7).

There could be various reasons why this student may be reluctant to communicate in English during class. It is possible that they feel self-conscious or embarrassed when speaking English in front of their classmates. A study conducted by Ref. [5], have shown that Ethiopian students experienced anxiety when speaking English. This fear of embarrassment could have a negative impact on their willingness to communicate in class. It is likely that students' reluctance to speak English in the classroom could also be due to negative experiences they may have had while learning the language. Negative learning experiences can manifest as any number of unpleasant situations that Ethiopian students may encounter while studying English in class, such as harsh correction, lack of support or encouragement, fear of making mistakes, boredom, lack of participation, and inadequate opportunities for speaking practice. Such negative experiences can lead to feelings of dissatisfaction with their learning, which in turn can cause students to be hesitant to participate in English communication and class discussions [5,6]. The teaching and assessment strategies employed by teachers also appear to be contributing to the students' reluctance to speak English in Ethiopian schools. According to the researcher conducted by Ref. [25], most English tests in Ethiopian schools focus more on reading and grammar skills than on developing speaking skills. This means that students may not be adequately assessed for their English communication skills, which can hamper their ability to speak English with confidence and fluency. In addition to these pedagogical factors, the cultural context of Ethiopia may also play a role in students' reluctance to speak English. With a diverse cultural landscape that includes numerous ethnic groups, languages, and traditions, students may feel a stronger attachment to their native language and culture. English may be viewed as a foreign language and a less important marker of its cultural identity and heritage. This preference for the native language can lead to a decreased motivation to actively engage in English communication.

Despite informal settings being conducive to providing a less stressful, safer, and more comfortable atmosphere for L2 conversation, opportunity to use English outside the classroom was few and far between. Ethiopian students who are learning English may be hesitant to communicate in English outside the classroom for various reasons. Limited exposure to English outside of the classroom may hinder their proficiency as they rarely encounter opportunities to use English in their daily lives, which may lead to a perceived lack of practical value. In addition, students may lack confidence in their English-speaking abilities and feel uncomfortable using English in real-life situations, fearing judgment, and making mistakes. Insufficient access to a variety of high-quality English language resources, such as digital tools and online content, can have a notable impact on students' ability and motivation to communicate effectively in English outside of the classroom. Without convenient access to authentic examples of English language use, including engaging digital platforms and online materials, students may struggle to find the necessary motivation to practice and enhance their English language skills.

Moreover, in Ethiopia, there are social and cultural factors that could discourage students from using English in their daily interactions. The culture of the country is largely shaped by collectivism and conformity. Collectivism prioritises the collective group, such as family or community, over individual interests. For Ethiopians, their identities are closely tied to their ethnic and language groups. Within a collectivist culture, people commonly experience a deep sense of belonging and accountability toward their community. The decisions and actions of the group take precedence over individual advantages. This cultural feature may influence the way language is used, particularly if the predominant language in the community is not English. It is possible for individuals to refrain from speaking English outside educational settings to avoid drawing attention to themselves or deviating from the linguistic conventions of their group. Furthermore, Ethiopians prioritise conformity and expect individuals to adhere to established social norms. This may impact language usage, as some individuals may be reluctant to speak English in a nonacademic environment if their community predominantly uses another language. Speaking English could be perceived as non-conformist behavior, leading to social scrutiny or pressure on those who do so. This situation is further compounded by the fact that government language policies and education systems may not prioritise the use of English beyond the classroom, which can decrease learners' motivation to engage with the language in their daily lives.

The self-assessed English proficiency of Ethiopian students learning English as a foreign language was found to be insufficient, based on their own evaluations of their ability to interact, produce, and speak the language. This self-awareness suggests that students recognise their own limitations in effectively using basic English. They may have difficulty expressing themselves clearly, comprehending English texts, or participating in basic conversations. These perceptions may arise from a variety of causes, including ineffective teaching and learning approaches, limited exposure to English-speaking environments, and a lack of practical opportunities to use the language beyond the classroom.

RQ2 How did the Ethiopian students' L2MSS, L2WTC inside and outside the classroom, and their self-assessed proficiency in English relate to one another?

The second research question addressed how students' L2MSS, L2WTC inside and outside the classroom, and their self-assessed English proficiency interacted with each other. Our findings indicated significant associations among the various components of L2MSS, which was consistent with the findings of previous studies [4,5,8,17]. When individual variables were examined more closely, the strongest statistically significant positive association was between the L2 learning experience and the ideal self. This indicated that students' future self-expectations would improve in direct proportion to the quality of their learning experience, and vice versa. The result was consistent with those found by Refs. [4,5,17] underpinning that students who have a positive outlook on their future as language users are more likely to value their L2 learning experiences than those who have a less favorable view of themselves as language users, as they fail to appreciate the value and purpose of what they experienced in their English classes. The study further revealed that there is a significant correlation between students’ ideal self and ought to L2 self, suggesting that people are more likely to strive towards their goals when they feel a sense of obligation to do so. Thus, the findings highlight the importance of creating a supportive and encouraging environment that motivates individuals to pursue their aspirations. In a study conducted by Zhou [8], it was discovered that applying a certain level of pressure can have a positive impact on students' mindset, causing them to set higher expectations for their future achievements. Based on the findings, there was also a positive and statistically significant correlation between the L2 learning experience and the ought to L2 self. The L2 learning experience encompasses aspects such as effective pedagogical approaches, conducive learning settings, and opprtunities for practical language application. This suggests that learners who undergo more positive, extensive, or fulfilling English language learning encounters generally have higher expectations or a stronger sense of obligation towards their language skills. Our findings correspond to a study conducted by Zhou [8], who demonstrated a positive correlation between the L2 learning experience and the ought to L2 self.

The two subscales for L2WTC (inside and outside the classroom) had a moderately significant positive correlation. The finding suggests that students who are comfortable engaging in English conversations within the classroom are more likely to utilize their language skills in real-life situations, and vice versa. This finding is in line with expectations based on Ethiopia's unique educational and environmental context. Unlike students in other countries with access to English-speaking communities and online resources, Ethiopian students rely mainly on their EFL classes for English practice and exposure. As a result, if they are reluctant to speak English in the classroom, their opportunities for language development and real-world language use are limited, which can lead to a reluctance to use English in authentic settings. In line with our findings, similar results were found by Refs. [4,7] indicating a positive correlation between classroom participation and willingness to use English in real-life scenarios. This highlights the crucial role of the classroom in fostering English language proficiency and emphasises the need for a supportive environment that encourages students to communicate both in and out of the classroom.

The three components of L2MSS displayed a significant positive correlation with L2WTC in the classroom. The result indicated a statistically significant positive association between the ideal L2 self of students and their L2WTC during class, as they strive to narrow the gap between their present and future selves. Essentially, students who possess a distinct and optimistic vision of themselves as proficient English speakers are more inclined to engage in classroom conversations and challenge themselves to enhance their language abilities. This underscores the significance of cultivating a robust sense of identity and motivation. The present study's findings corroborate the results of previous studies, such as[4,7,17], which revealed a positive and statistically significant positive relationship between the ideal L2 self and L2WTC in a classroom setting. The statistically significant positive correlation between ought to L2 self and L2WTC in classroom settings suggests that students who are concerned about their ought to L2 self are more likely to engage in classroom interactions. In this regard, external pressures, obligations, and expectations associated with ought to L2 self may have a beneficial impact on L2WTC within the classroom. Our results aligned with Lee & Lee [7], indicating a positive correlation between L2 ought to self and L2WTC in the classroom setting. However, Peng [4] did not observe a statistically significant link between the two in the classroom. These findings suggested that the L2 ought to self may be relevant but require further investigation. We also found a statistically significant positive correlation between the L2 learning experience and the L2WTC in class, echoing the belief that supportive learning environments promote communication [3,4]. Our findings indicate that positive classroom experiences, such as the presence of engaging and responsive educators, opportunities for collaborative peer interactions, a communication-focused curriculum, an inclusive classroom culture, active parental involvement and support, access to educational resources, and positive reinforcement, can greatly enhance students' L2WTC and overall success.

The results of our study also indicated that the three components comprising L2MSS demonstrated a significant and positive correlation with L2WTC outside the classroom. This association suggests that an increase in the three components of L2MSS, namely ideal L2 self, ought-to L2 self, and positive learning experiences, results in a corresponding increase in L2WTC in non-classroom settings. Students who possessed a well-defined and positive ideal L2 self, meaning a clear perception of themselves as competent language users in the future, exhibited a greater propensity to engage in communicative interactions in English beyond the confines of the classroom. The rationale behind this inclination is linked to the motivational influence of their optimistic self-image as an eventual proficient speaker. Students who held a sense of duty or were subject to external pressures to perform well in their language acquisition endeavors (i.e., ought to L2 self) exhibited a higher likelihood of participating in L2WTC outside the classroom. Such external expectations, which could originate from teachers, peers, or family members, served as a driving force for students to proactively utilize the language. Engaging and interactive classroom activities, effective teaching methods, supportive learning environments, and opportunities for meaningful language practice have been found to contribute positively to an increase in L2WTC outside the classroom. Research indicates that when students enjoy and benefit from language learning, they are more likely to apply their new skills in real-world scenarios. This underscores the value of fostering an enjoyable and effective learning environment that can have a positive impact on a student's language development. The outcome that the three components comprising L2MSS demonstrated a significant and positive correlation with L2WTC outside of the classroom offers further credence to the conclusions of earlier research in other EFL settings, such as Korea [7] and China [8]. These investigations underpin the statistically significant positive relationships among the three components of the L2MSS and L2WTC outside the classroom.

Our research has shown a significant link between how students rate their own English language proficiency and their willingness to communicate in English. Specifically, students who perceive themselves as having strong English skills are more likely to engage in the language. This observation is not surprising, given that one's confidence in one's communication abilities plays a vital role in their willingness to interact in a foreign language. When a student is confident about their English proficiency, they are more likely to communicate fluently and competently [2]. However, in contrast, students who lack confidence in their language skills may experience language anxiety, which can hinder their ability to communicate effectively. The findings were in line with previous studies conducted by Refs. [6,18]. According to the correlation analyses conducted by Peng [4], perceived communication skills were found to have a strong association with L2WTCoutside the classroom. Similarly MacIntyre et al. [22], reported a significant positive correlation between perceived communication competence and LTWTC both inside and outside the classroom. This underscores the importance of building self-efficacy and confidence in language learning and highlights the need to cultivate positive self-perceptions to boost students' motivation and ability to use the language effectively.

Through our research, we have found a strong and positive correlation between the three components of L2MSS and students' self-reported level of English proficiency. Our findings indicated that students who hold a strong belief in their ideal L2 self, feel obligated to improve their English skills, and have positive learning experiences, were more likely to rate their English proficiency higher. This correlation between motivation and English proficiency is noteworthy, as it highlights the importance of a positive mindset, a sense of responsibility, and enjoyable learning experiences for students to evaluate their English proficiency positively. Our research findings were in parallel with those those of Roshandel et al. [19], where a statistically significant correlation was found between the components of L2MSS and L2 self-efficacy. However, our results contradict those of Subekti [9], which found no statistically significant relationship between achievement and any of the L2MSS components. The discrepancy in these findings can be attributed to the indirect relationship between motivation and actual learning outcomes, as argued by Ref. [11]. According to Csizér and Dörnyei[11], motivation serves as a precursor to action rather than a measure of success itself. Therefore, studies that focus only on the direct association between motivation and L2 achievement measures, such as course grades, may have overlooked the intermediary link called motivation drive.

RQ3 What was the predictive effect of students' L2MSS on their self-assessed English proficiency and L2WTC inside and outside the classroom?

Our study analyzed how students' L2MSS predict their L2WTC and English proficiency ratings.. By calculating the path coefficients, we were able to determine the impact of each independent variable on the dependent variables.

Initially, we evaluated the influence of the ideal L2 self, L2 learning experience, and ought to L2 self on students' self-assessed English proficiency. The results showed that each of these components had statistically significant positive predictive effects on self-rated English proficiency. Specifically, having a positive ideal L2 self, a favorable L2 learning experience, and trying to meet obligations and others' expectations were all strong predictors of L2 learners' self-assessed English proficiency. Additionally, the R^2^ value was 0.332, indicating that the three components of the L2MSS accounted for 33.2 % of the variation in how well people perceived their English proficiency. So, the three parts of the L2MSS were found to be important indicators of how well L2 learners thought they spoke and understood English. The results of our study agree with those of studies [10,19], which found that the L2MSS components make people feel better about their own abilities. However, our results contradict the findings reported by Ref. [9]. Despite the widely accepted belief among experts that students' L2MSS can predict L2 learning outcomes, Subekti [9] found no significant correlation between the participants' L2MSS and their EFL achievement. These disparities could be attributed to the use of different achievement indicators.

Next, we assessed the impact of L2MSS and self-assessed English proficiency on L2WTC inside the classroom. The study has shown that the factors of ideal L2 self, ought-to L2 self, L2 learning experience, and perceived English proficiency positively affect students' willingness to use English in the classroom. The study's R^2^ value, 0.460, indicates that these four variables collectively explain 46 % of the variation in students' L2WTC during class. In our study, having a clear vision of one's ideal L2 self, feeling a sense of obligation to improve one's language skills, having positive experiences with language learning, and perceiving oneself as proficient in English were found to predict L2WTC inside the classroom positively. The results of the study were in line with the findings previously reported by Refs. [10,17,19]. According to Sadoughi & Hejazi[17], WTC in the classroom was positively influenced by the ideal self L2, ought-to-self, and learning experience. On the other hand [10,19], reported that the different elements of L2MSS had a significant impact on self-efficacy.

Finally, our study aimed to assess the impact of the L2 learning experience, the L2 self, and self-assessed English proficiency on L2WTC outside the classroom. The results showed that L2 learning experience, ought to L2 self, and self-assessed English proficiency had a positive effect on L2WTC outside the classroom, with an R^2^ value of.380. This implies that the three variables together accounted for 38 % of the variances in L2WTC outside the classroom. These findings are consistent with the results reported in previous studies [6,7], and [8]. According to Nagy [6], self-perceived English communication ability strongly and positively predicted L2WTC outside the classroom. Additionally, [7,8] reported that the ought to L2 self and L2 learning experience, respectively, positively predicted L2WTC outside the classroom.

## 6 Conclusion

The study examined the L2 motivational self-system, willingness to communicate, and self-assessed English proficiency of 12th-grade Ethiopian EFL students and analyzed the relationships and predictive effects among these variables. The study provides insights into the L2MSS of Ethiopian students, their L2WTC in and outside the classroom using L2, and their self-assessed proficiency in English. The findings showed that Ethiopian students were of low interest in using English, lack confidence in utilizing all four language skills, and exhibit minimal levels of communication inside and outside the classroom.

The empirical evidence indicating that students' ideal L2 self and L2 learning experience were below average while their ought-to L2 self was high has profound research implications. This observation underscores the existence of a significant disparity between the students' envisioned future selves as proficient English speakers (ideal L2 self) and their current perceptions of their language abilities and learning experiences. Students may feel anxious or frustrated due to the perceived gap between their desired language proficiency and their current abilities [3]. Teachers should address these concerns by providing support, guidance, and opportunities for students to set realistic goals, monitor their progress, and celebrate their achievements. Digital technology can also help learners imagine and visualize their future selves as part of an English-speaking community [12]. Online platforms and resources provide access to authentic English language materials, connections with native speakers or proficient users, and virtual language communities. These digital tools and interactions boost learners' motivation to learn and communicate in English by assisting them in envisioning themselves as part of an English-speaking community. Moreover, digital technology's accessibility and convenience enable learners to engage in language learning at any time and from any location, making it easier for them to actively pursue their language goals and develop their ideal L2 self. The presence of a strong sense of obligation to improve their English skills, known as high ought-to L2 self among students, indicates that external factors, such as societal pressure and expectations, have a significant impact on their motivation to learn English. It is important for educators to be aware of these external influences and create a supportive learning environment that balances students' sense of duty with intrinsic motivation and personal interest in language learning. Creating a positive and supportive classroom environment, using engaging and interactive teaching methods, providing meaningful and relevant learning materials, fostering active participation and communication, offering timely and constructive feedback, differentiating instruction to meet individual needs, and promoting a growth mindset can all help teachers improve students' learning experiences [15]. These strategies can enhance students' motivation, engagement, and language development, leading to a more effective and enjoyable learning experience.

Our research has shown that the usage of English language by students, both inside and outside the classroom, is extremely low. This is a cause for concern, as English is an important language for communication in today's globalised world, and it could potentially affect the future job prospects of these students. Even if students live in an area where the English language is not commonly spoken outside the classroom, it is essential that teachers create a conducive environment that motivates students to utilise digital technologies such as YouTube, email, and other social media platforms to communicate better. Teachers can leverage these digital tools to create an interactive learning environment that fosters a willingness to communicate in English both inside and outside the classroom. Online discussion forums, video conferences, and language learning applications can be used to promote collaboration between students, thereby enhancing their communication skills in English. Furthermore, the Ethiopian government should play an important role in expanding the necessary digital technology infrastructure. This can be achieved by investing in infrastructure development, providing access to digital devices and reliable internet connectivity, and developing localised digital resources that can help support English language learning. The government can also provide training and professional development opportunities for teachers to enhance their digital literacy skills and effectively integrate technology into their instructional practices. The low self-assessed English proficiency of Ethiopian students is a crucial issue that demands immediate action. Teachers can play a vital role in assessing language skills accurately and providing constructive feedback to help students develop a realistic perception of their proficiency. The findings highlight the significance of providing targeted support and feedback, effective teaching methods, and continuous professional development.

Within the Ethiopian context, the results indicated that the three elements of the L2MSS (ideal L2 self, ought to L2 self, and learning experience) had a positive and statistically significant influence on both L2WTC and self-assessed English proficiency. These findings have important implications for teaching practices in this context. First, teachers should prioritize the development of their students' ideal L2 self through the provision of exemplars and the demonstration of the advantages that English proficiency can offer in terms of future academic and professional prospects. Second, engaging and interactive language learning activities that incorporate Ethiopian literature, history, and cultural content can enhance students' motivation and proficiency in English language learning. Third, teachers can improve their students' English language skills by providing them with a set of strategies, such as building their vocabulary, using techniques to enhance listening comprehension, and practicing speaking. Besides, it is important to encourage students to take responsibility for their own learning process and reflect on their progress. By doing so, teachers can help boost students' confidence and motivation to communicate effectively in English [22]. Finally, empowering students and enhancing their motivation can be achieved through self-assessment, goal setting, and self-reflection. Teachers can assist students in setting achievable goals, monitoring their progress, and celebrating their achievements, which fosters autonomy and self-efficacy.

## Limitations and future research

There is still considerable room for improvement, even though the findings of this research shed light on the interrelatedness between L2MSS, L2WTC in and outside the classroom, and self-assessed English proficiency. Cross-sectional data for this research was obtained from students at a large preparatory school. Although the sample size was large, they should be interpreted with care, as they involved students at one school. Future studies would benefit from including more participants from a variety of demographic and geographical backgrounds, and longitudinal studies would offer further insights into processes. The study presented in the research paper primarily focuses on quantitative data and doesn't delve deeply into the qualitative aspects of students' experiences and perceptions. To gain a more profound understanding of the underlying factors influencing students' motivation and communication in English, future research could employ qualitative methods, such as interviews or focus groups.

## Data availability

It is crucial to note that the data and information under reference are still undergoing analysis or review, as they form part of an ongoing investigation. It is of utmost importance to maintain the integrity and fairness of the investigation by keeping the information confidential until the investigation is complete and official conclusions are made.

## Funding statement

This work was supported by the Open Access Fund of the University of Szeged (grant number: 6094).

## CRediT authorship contribution statement

**Merih Welay Welesilassie:** Writing – original draft, Validation, Project administration, Methodology, Investigation, Formal analysis, Data curation, Conceptualization. **Marianne Nikolov:** Writing – review & editing, Supervision.

## Declaration of competing interest

The authors declare that they have no known competing financial interests or personal relationships that could have appeared to influence the work reported in this paper.
